# Structural insights into the ability of nucleoplasmin to assemble and chaperone histone octamers for DNA deposition

**DOI:** 10.1038/s41598-019-45726-7

**Published:** 2019-07-01

**Authors:** Aitor Franco, Rocío Arranz, Noelia Fernández-Rivero, Adrián Velázquez-Campoy, Jaime Martín-Benito, Joan Segura, Adelina Prado, José M. Valpuesta, Arturo Muga

**Affiliations:** 1 Instituto Biofisika (CSIC, UPV/EHU) and Departamento de Bioquímica y Biología Molecular, Facultad de Ciencia y Tecnología, Universidad del País Vasco (UPV/EHU), Aptdo. 644, 48080 Bilbao, Spain; 2 Departamento de Estructura de Macromoléculas, Centro Nacional de Biotecnología (CNB-CSIC), Campus de la Universidad Autónoma de Madrid, 28049 Madrid, Spain; 3 Institute of Clinical Sciences, Faculty of Medicine, Imperial College London, W12 0NN London, United Kingdom; 4 Medical Research Council, London Institute of Medical Sciences, W12 0NN London, United Kingdom; 5 Institute of Biocomputation and Physics of Complex Systems (BIFI), Joint Units IQFRCSIC-BIFI, and GBsC-CSIC-BIFI, Universidad de Zaragoza, 50018 Zaragoza, Spain; 6 Department of Biochemistry and Molecular and Cell Biology, Universidad de Zaragoza, 50009 Zaragoza, Spain; 7 Aragon Institute for Health Research (IIS Aragon), 50009 Zaragoza, Spain; 8 Biomedical Research Networking Centre for Liver and Digestive Diseases (CIBERehd), 28029 Madrid, Spain; 9 Fundacion ARAID, Government of Aragon, 50018 Zaragoza, Spain

**Keywords:** Biophysical chemistry, Cryoelectron microscopy

## Abstract

Nucleoplasmin (NP) is a pentameric histone chaperone that regulates the condensation state of chromatin in different cellular processes. We focus here on the interaction of NP with the histone octamer, showing that NP could bind sequentially the histone components to assemble an octamer-like particle, and crosslinked octamers with high affinity. The three-dimensional reconstruction of the NP/octamer complex generated by single-particle cryoelectron microscopy, revealed that several intrinsically disordered tail domains of two NP pentamers, facing each other through their distal face, encage the histone octamer in a nucleosome-like conformation and prevent its dissociation. Formation of this complex depended on post-translational modification and exposure of the acidic tract at the tail domain of NP. Finally, NP was capable of transferring the histone octamers to DNA *in vitro*, assembling nucleosomes. This activity may have biological relevance for processes in which the histone octamer must be rapidly removed from or deposited onto the DNA.

## Introduction

Histone chaperones are members of a complex protein network that prevent undesired nonspecific interactions between highly basic histones and nucleic acids or other cellular components. They differ in structure, histone cargo and binding mechanism, and are involved in histone synthesis, storage, exchange and transport from the cytoplasm to the nucleus, presentation for post-translational modification (PTM) and nucleosome assembly/disassembly^1,2^. All these processes are essential for maintenance of chromatin integrity, DNA replication and repair, and gene activation and transcription.

**Figure 1:**
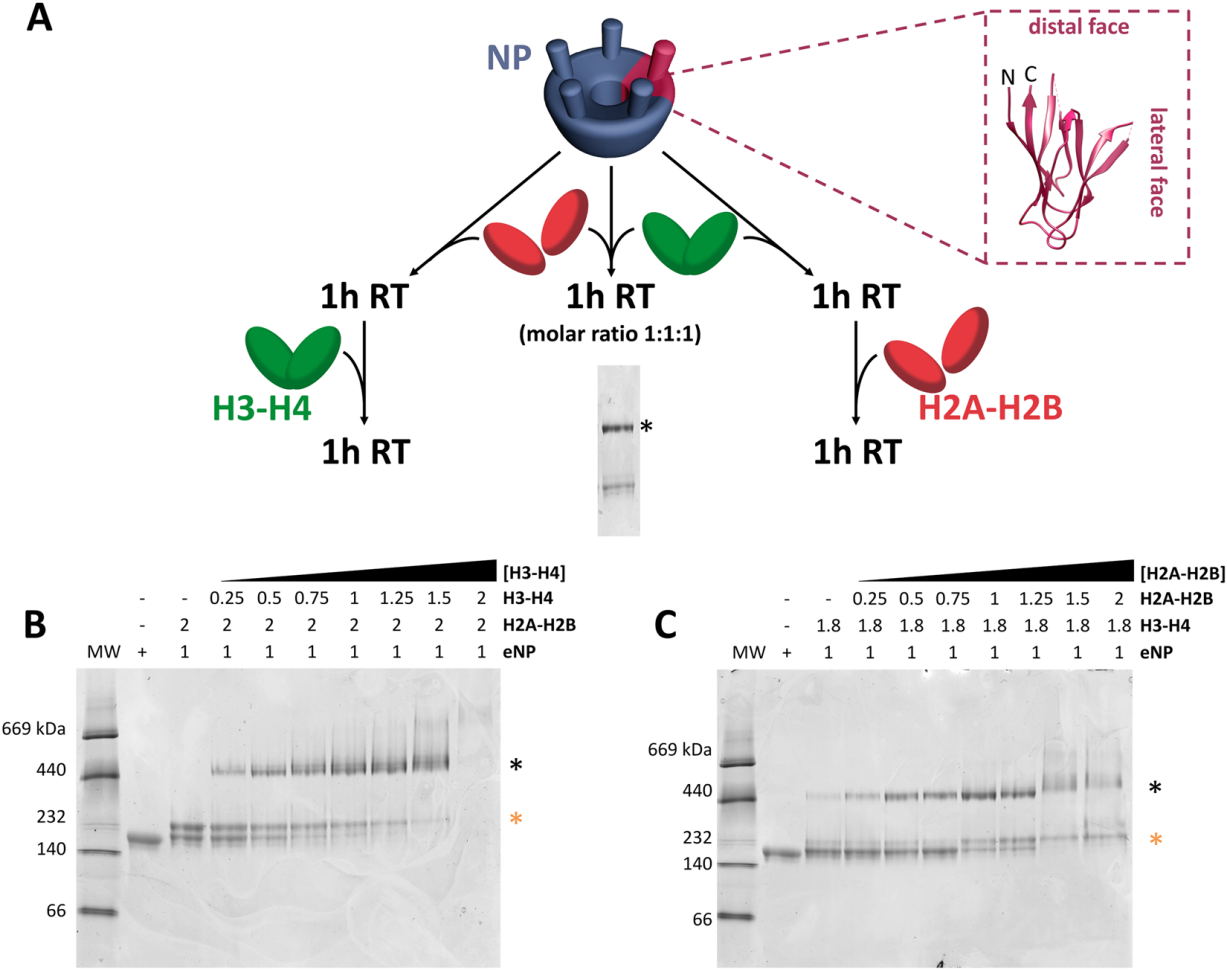
Nucleoplasmin (NP) assembles the histone octamer. (**A**) Egg NP (eNP; from *X. laevis*) was incubated with either H2A-H2B or H3-H4 and the formed eNP/H2A-H2B or eNP/H3-H4 complexes were titrated with H3-H4 (**B**) and H2A-H2B (**C**), respectively. Samples were analyzed by 4–16% native PAGE and stained with Coomassie Brilliant Blue. The complex formed by two NP pentamers and a histone octamer is denoted with a black asterisk, and is also formed when both dimers were added at once (gel image from A). The complexes of NP with either H2A-H2B or H3-H4 dimers are marked with an orange asterisk. Inset: crystal structure (pdb 1K5J) of a monomeric NP core domain.

Nucleoplasmin (NPM2 or NP) is a member of the histone chaperone nucleophosmin/nucleoplasmin family, which is present throughout the animal kingdom. NP is a homopentameric, acidic and thermostable protein capable of promoting nucleosome assembly from purified DNA and histones^3,4^. It plays a crucial role in sperm chromatin decondensation after fertilization^5–7^, nucleolus assembly in mouse embryos^8^, modulation of activity of the chromosomal passenger complex, a regulator of cell division in *Xenopus*^9^, and somatic chromatin remodeling during replication licensing and cell reprogramming^10,11^. NP monomers (200 residues) are divided into an N-terminal core domain (residues 1–120) and a C-terminal tail domain (121–200). The core domain folds into an eight-stranded β-barrel with a jelly-roll topology (Fig. 1A inset), contains a short acidic tract (A1), and is responsible for NP oligomerization^12–14^. In contrast, the tail domain adopts an intrinsically disordered conformation, and contains two acidic segments (A2 or polyGlu; and A3) and a basic bipartite nuclear localization sequence (NLS). This domain is located at one extreme of the pentamer known as the distal face, which is involved in histone binding^15,16^. PTMs, in particular phosphorylation, activate the chromatin decondensation activity of NP^6,17–20^, enhancing its ability to remove linker histones from DNA^7^. NP has classically been considered a histone chaperone for H2A-H2B dimers. Under saturating conditions, one NP pentamer binds five histone dimers at its distal face^15,21,22^. This interaction has been associated with storage of H2A-H2B dimers in oocytes and their sequential addition onto DNA to assemble the nucleosome after fertilization. Immunoprecipitation assays using *Xenopus* oocytes and egg extracts have also suggested that NP may also chaperone H3-H4 *in vivo*^16^. Electron microscopy has shown that two NP pentamers, facing each other through their distal face, wrap the H3-H4 histone in the center of the particle, with a stoichiometry of 2/2 (NP pentamer/histone tetramer)^16,22^. This ellipsoidal complex is only observed with H3-H4 tetramers and not when the histone association equilibrium is shifted towards the dimer^16^. Several studies agree that NP is able to assemble large complexes that contain the four core histones. Two binding models for the interaction of NP with histone octamers have been proposed. In the first one, the lateral face of a NP pentamer would first interact with H2A-H2B dimers and the subsequent binding of H3-H4 tetramers would trigger association of two pentamer/histone dimer complexes. Thus, the NP decamers would create on their lateral face a binding region for H2A-H2B dimers and H3-H4 tetramers that could assemble into histone octamers on the chaperone^12–14,23^. In the second model, pentameric NP binds a histone octamer equivalent consisting of equal amounts of the four core histones, not necessarily adopting an octamer structure^24^.These models were put forward based on experimental data obtained with recombinant NP, and therefore the effect of post-translational modifications (PTMs) of NP in histone binding could not be taken into account. Our previous work suggests that interaction of the natural, hyperphosphorylated eNP with the histone octamer gives rise to ellipsoidal complexes similar to that found for H3-H4, in which two eNP pentamers sandwiched an intact histone octamer^22^. Whereas the interaction of NP with H2A-H2B and H3-H4 has been characterized in detail, complex formation with histone octamers awaits a more in depth characterization.

We explore in this work the structural bases of the interaction between NP and stable histone octamers, and the ability of NP to sequentially bind nucleosomal histones to assemble the histone octamer. Our data show that besides interacting with the complete octamer, NP also assembles the histone octamer from its constituents. The sequential interaction of the chaperone with H2A-H2B and H3-H4 generates and stabilizes an octamer that adopts a nucleosome-like structure. This was confirmed by the 3D reconstruction of the NP/octamer complex by cryoelectron microscopy (cryoEM), showing that the two NP pentamers interact with the histone octamer in an asymmetric manner, with a stronger binding on the H2A-H2B face of the octamer, a region with a high density of positively charged residues. To further understand the mechanism involved in this interaction, we studied the interaction between isolated tail domains and octamers, revealing that the octamer binds 4 tail domains. This finding suggests that cooperation between several protomers is required for octamer binding. Finally, nucleosome assembly was observed when stable NP/octamer complexes were challenged with DNA, indicating that NP mediates transfer of the full octamer to DNA.

## Results

### eNP can interact with the complete octamer or sequentially bind the octamer components to assemble an octamer-like structure

**Figure 2:**
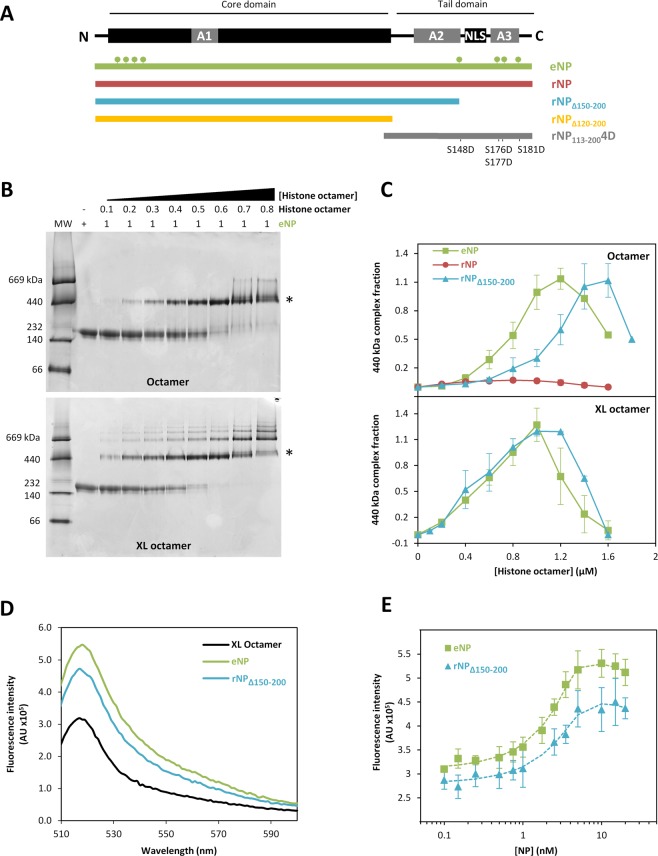
Phosphorylation and exposure of the acidic tract regulates nucleoplasmin (NP)/histone octamer complex formation. (**A**) Schematic representation of the recombinant NP (rNP) variants used in this study. The phosphorylation sites (green circles) of egg NP (eNP; from *X. laevis*), the position of the acidic tracts in both protein domains (A1–3), and the location of the nuclear localization sequence (NLS) are shown. (**B**) Titration of native histone octamers (top panel) and crosslinked octamers (XL octamer, bottom panel) with eNP. Samples were analyzed by 4–16% native PAGE and stained with Coomassie Brilliant Blue. The complex formed by two NP pentamers and a histone octamer is marked with an asterisk. (**C**) Quantification of the NP/octamer complex marked with an asterisk in (**B**). The 440 kDa complex fraction was estimated as the ratio of the intensity of the NP/octamer complex band and that of eNP (squares), rNP (circles) or rNP_Δ150–200_ (triangles) in the absence of histones. Solid lines connecting the symbols have no physical meaning and are just to guide the eye. (**D**) Fluorescence spectra of the histone octamer (H2BT71C-Alexa 488) in the absence (black) and presence of eNP (green) or rNP_Δ150–200_ (blue). **(E)** Titration of the histone octamer (H2BT71C-Alexa488) with eNP (squares) or rNP_Δ150–200_ (circles). Histone concentration was 5 nM and NP concentration is given for the pentamer. Data are shown as mean ± s.d. of at least three different experiments. Data for rNP_Δ150–200_ have been shifted in the y-axis for the sake of clarity.

We previously demonstrated that NP can bind histone octamers and that this interaction can be followed by native electrophoresis, as complex formation generates a characteristic 440 kDa band^16^. These experiments were carried out by diluting the octamer from 2 M NaCl, which stabilizes the oligomer, to a physiological ionic strength that allows the interaction. Since the histone octamer can disassemble before binding to the chaperone in these conditions, the actual molecular basis of the interaction was not clear. Thus, our first aim was to find out if sequential interaction of the chaperone with the core histones H2A-H2B and H3-H4 leads to octamer assembly. To answer this question, complexes of the natural phosphorylated variant, eNP, and either H2A-H2B or H3-H4 were mixed with the other counterpart core histones (i.e., H3-H4 or H2A-H2B, respectively) (Fig. 1A), and the result analyzed by native PAGE (Fig. 1B,C). As previously reported^15^, binding of two H2A-H2B dimers to an eNP pentamer generated a complex of around 232 kDa, whose intensity decreased with increasing H3-H4 concentrations, with the concomitant appearance of a high molecular weight band (Fig. 1B). The reverse titration, e.g., eNP/H3-H4 (1/2) complexes challenged with increasing concentrations of H2A-H2B dimers, generated the same 440 kDa band characteristic of an octamer-like conformation in complex with the chaperone (Fig. 1C). In this case, the 440 kDa band was also detectable in the presence of only H3-H4 (Fig. 1C, lane 3). This difference was due to the association equilibrium of H3-H4 that, in contrast to H2A-H2B, involves dimers and tetramers^25^, being two tetramers capable of forming a complex with the distal face of two NP pentamers^16^. In both titrations, at an eNP/H2A-H2B/H3-H4 molar ratio of 1/1/1 (equivalent to 1/0.5 NP/octamer), most of the low molecular weight NP/histone complex disappeared and the 440 kDa complex, which contained the four nucleosomal histones and NP (Supplementary Methods and Fig. S1), became the major band. The same complex was observed when both core histones were added simultaneously to eNP (see gel in Fig. 1A). To further test the ability of NP to bind the octamer, we compared the complexes formed between eNP and either native or crosslinked histone octamers (Fig. 2B). The use of crosslinked octamers ensured that this ligand did not disassemble due to experimental conditions (Fig. S2). eNP formed the high molecular weight complex (Fig. 2B) observed previously with both ligands^16^ (Fig. 1). The decrease of the 440 kDa band intensity observed in Fig. 2C at high histone concentrations could come from formation of larger complexes, more evident with crosslinked octamers (Fig. 2B; lower panel) and/or partial sample aggregation (Fig. 2B), as previously seen with other histone chaperones^26^. These complexes might arise from alternative interactions between crosslinked octamers and NP, especially at high histone concentration, as the addition of octamers to the 440 kDa complex. This electrophoretic pattern was not observed with native, non-crosslinked octamers, and only a very weak 669 kDa band was seen at eNP/octamer molar ratios higher than 1/0.6. Together, these results demonstrate the ability of NP to sequentially bind both core histone dimers, facilitating their assembly into an octamer-like conformation, and to interact with preformed histone octamers.

Post-translational modifications of NP and the flexible C-terminal tail domain, particularly exposure of the acidic polyGlu tract, play a crucial role in NP histone binding activity^6,15–17,19,20,22^. Therefore, we also investigated their relevance in NP/histone octamer complex formation. For this purpose, we compared the interaction of native, hyperphosphorylated eNP and three recombinant peptides: full-length rNP and the truncated mutants rNP_Δ150–200_ and rNP_Δ120–200_ (Fig. 2A), with histone octamers. Comparison between full-length rNP and eNP allowed us to estimate the effect of post-translational modifications on the interaction, and the use of different NP deletion mutants, to identify the protein regions engaged in complex formation. Although rNP could not efficiently form a stable complex with native histone octamers (Fig. S3A), highlighting the importance of PTMs, it bound the crosslinked ligand forming a complex with a higher apparent molecular mass (approx. 500 kDa; Fig. S3B). This larger complex could arise from the requirement of more NP pentamers to neutralize the basic ligand. Deletion of the last 50 amino acids of this domain, which enhances exposure of the acidic tract, restored the chaperone´s capacity to form a complex similar to that found for eNP (Fig. S3C,D, Fig. 2C), whereas the NP variant lacking the last 80 residues (and thus the polyGlu acidic tract), rNP_Δ120–200_, did not form this type of complexes with either native (Fig. S3E) or crosslinked octamers (Fig. S3F).

The apparent affinity of eNP and rNP_Δ150–200_ for Alexa-labeled, crosslinked octamers was estimated by fluorescence spectroscopy, as octamer binding to NP induced an increase in fluorescence intensity (Fig. 2D). The K_d_ values for the interaction of eNP and rNP_Δ150–200_ with crosslinked octamers were 0.04 ± 0.01 and 0.27 ± 0.06 nM, respectively (Fig. 2E). These values demonstrate that NP can bind preformed octamers with high affinity, similar to that described for their isolated histone components^22^, and that the tail domain regulates the interaction. Taken together, these data indicate that, as it was previously found for other NP/histone complexes^15,16,20,22,27,28^, PTMs of NP and exposure of the acidic A2 tract at the intrinsically disordered C-terminal arms of the chaperone are essential to embrace the histone octamer and stabilize the complex under conditions that favor octamer dissociation.

### 3D reconstruction of the NP/octamer complex

**Figure 3:**
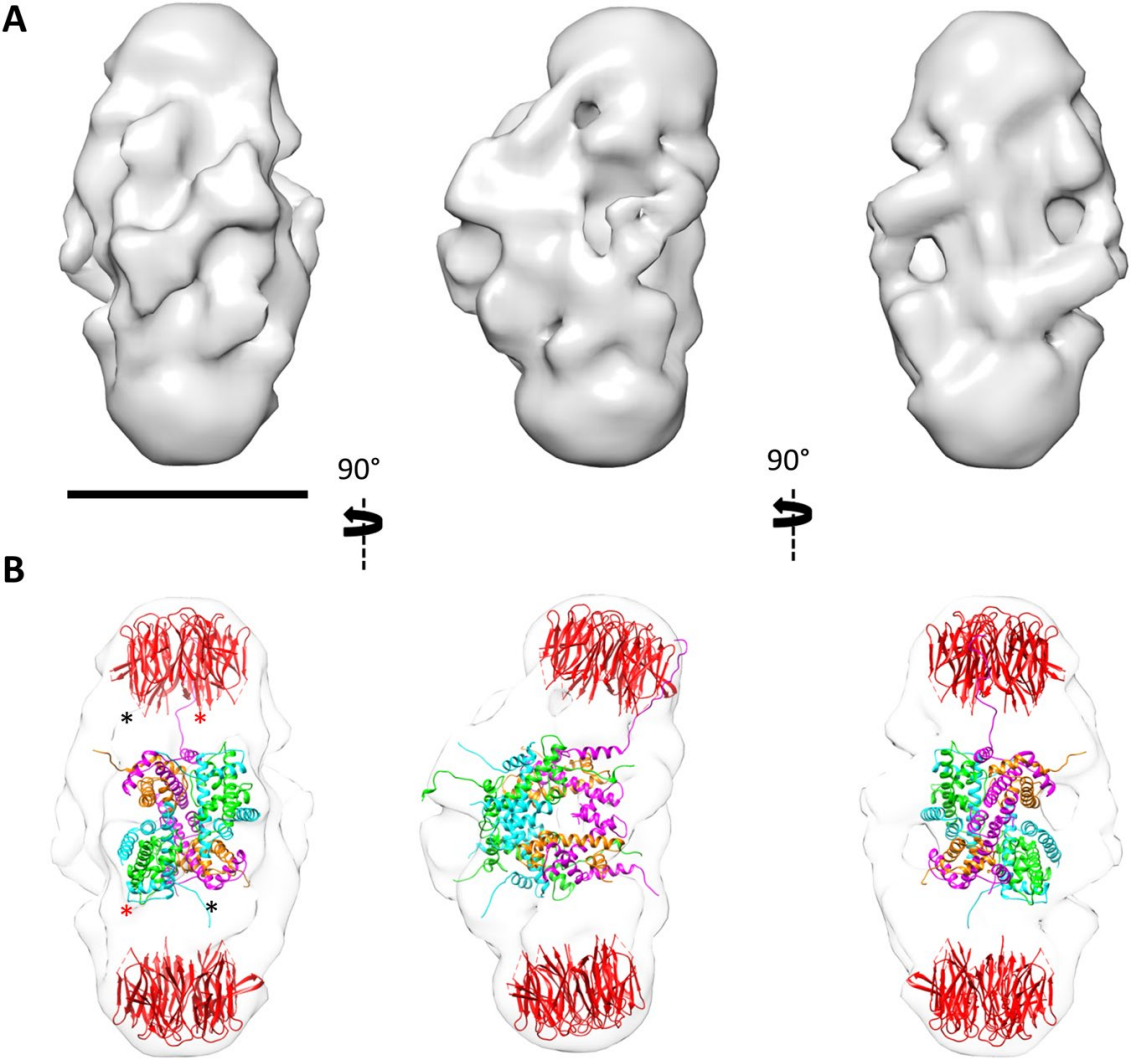
CryoEM structure of the egg nucleoplasmin (eNP)/octamer complex. (**A**) Three orthogonal views of the eNP/octamer complex; bar = 100 Å. (**B**) The same three views with docking of the core domain of NP (pdb 1K5J; colored in red) in the two extremes of the complex, and the histone octamer (extracted from the structure of the nucleosome; pdb 1AOI), in the center of the 3D reconstruction. The two copies of H2A are colored in green, H2B in light blue, H3 in magenta and H4 in gold. The asterisks highlight the tails of the pentameric chaperone that interact with the base of the octamer. The other three tails are spread along the rest of the octamer structure. The reliability of the docking is depicted in Fig. S5, where the components of the complex are fitted into a map at a 4σ threshold.

We then sought to determine the structure of the eNP/octamer complex using cryoEM. Aliquots of the complex were vitrified and movies were recorded on a Titan Krios equipped with a Falcon II detector, as described in the Methods section. A total of 166,976 particles of the complex were selected, after movie and CTF correction, and subjected to 2D classification (Fig. S4). All the classes showed an elongated, symmetrical structure with three strong mass densities, two at the extremes and one at the center, which was interpreted as two NP pentamers embracing the histone octamer^16^. However, the classes also showed a clear heterogeneity associated with the central mass and with the length of the particle (see below). The most populated class (11,438 particles) was then subjected to a 3D reconstruction procedure that revealed a structure with an apparent two-fold symmetry. To reinforce the 3D reconstruction, D1 symmetry was imposed. The final volume showed a slightly curved structure ~19 nm in length and ~10 nm wide at its central part (Fig. 3A). Docking of the atomic structure of the NP core (pdb 1K5J) at the two ends of the 3D reconstruction, and of the histone octamer (pdb 1AOI) in the central part was straightforward (Fig. 3B), and not only confirmed the location of the three oligomers in the complex, but in the case of the octamer clearly pointed to a structure very similar to that forming the nucleosome. Images of Fig. S5A, which depicts this complex at a 4σ threshold (displaying the regions of the complex with the highest density), clearly showed the good fitting of the octamer structure into the central part of the NP/octamer complex. They also suggest that the octamer sandwiched between the two NPs has a similar conformation to that found in the nucleosome structure. The use of the histone octamer structure in the docking is also supported by FRET experiments, which showed that NP-bound octamer components adopt a structure similar to that found in 2 M NaCl and in the nucleosome^16,29^. The stoichiometry of this complex is further reinforced by previous equilibrium sedimentation studies that according to its estimated molecular mass of 307 kDa, suggested that two eNP pentamers could associate with a histone octamer^16^.

As described above, the structure of the NP/octamer complex is curved, caused by the NP pentamers interacting with the histone octamer at a ~20° angle with respect to the longitudinal axis of the complex (Fig. S5A). The rest of the reconstructed mass of the NP/octamer complex could be assigned to the tail of the NP monomers (the last 80 residues), a flexible and negatively charged region that is known to be disordered but critically involved in the interaction with both types of core histones, H2A-H2B and H3-H4^15^ and the histone octamer^16^ (Figs S3 and S5). The resolution of the 3D reconstruction only allowed a partial localization of these disordered tails, but the map nevertheless showed that the tails of the NP monomers were arranged asymmetrically around the rectangular shape of the octamer. Two tails of each pentamer seemed to interact with the base of the histone complex (see asterisks in (Fig. 3B). This is the main interacting region between the chaperone and the octamer, and according to the docking it is localized in the H2A-H2B region, at the core of the octamer structure (Fig. 3B). This coincides with the part of the octamer that is protected from proteolysis when complexed to NP (Fig. S5B)^16^ and with the region that contains the largest number of positively charged residues that interact with the negatively charged amino acids of the NP C-terminal region (Fig. S5C).

### Cooperativity between different NP protomers is required for complex formation with the histone octamer

The stoichiometry of the eNP/histone complex is different for the distinct types of nucleosomal histones, as a NP pentamer binds five H2A-H2B dimers in a cup-like structure, whereas an ellipsoidal particle is formed with two H3-H4 tetramers or one histone octamer sandwiched by two chaperone pentamers^16,22^. Despite these differences, the chaperone employs the same region, the distal protein face where the flexible acidic tails are located, to interact with all histones.

**Figure 4:**
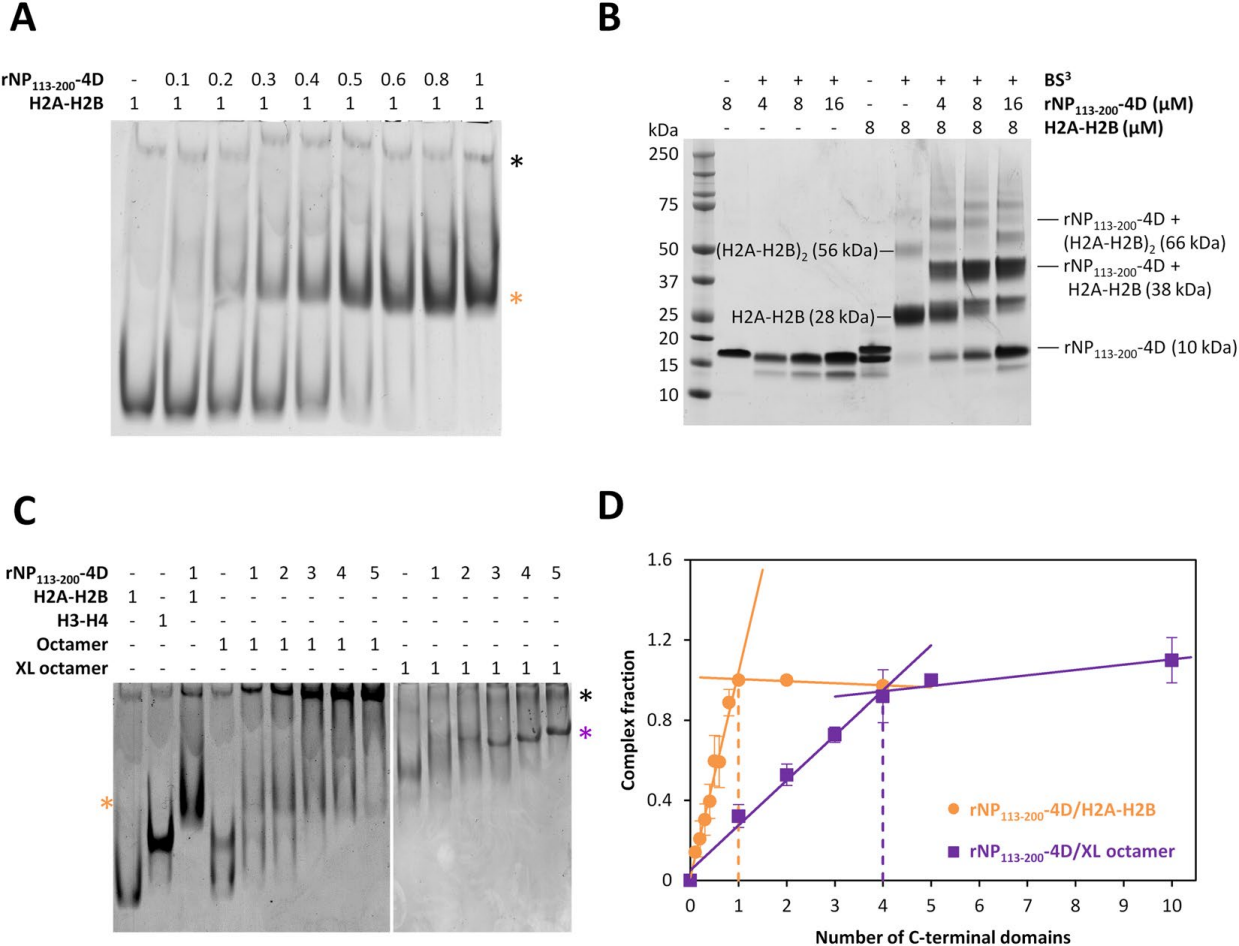
Cooperation of several nucleoplasmin (NP) C-terminal domains is required to bind the histone octamer. (**A**) H2A-H2B was incubated with increasing amounts of rNP_113–200_-4D for 1 h, samples were analyzed by 8% native PAGE and stained with Coomassie Brilliant Blue. (**B**) H2A-H2B/rNP_113–200_-4D complexes were crosslinked with bis(sulfosuccinimidyl)suberate (BS^3^), analyzed by denaturing NuPAGE Novex 4–12% Bis-Tris gel in MES buffer and stained with Coomassie. Bands are labeled with the stoichiometry inferred from their apparent molecular weight and their theoretical molecular weight in brackets. (**C**) The interaction between rNP_113–200_-4D and native or crosslinked histone octamers was studied by 8% native PAGE as in (**A**). **(D)** Saturation stoichiometries were estimated as the molar ratio of tail domain/histone H2A-H2B dimer (orange circles) or tail domain/crosslinked octamer (purple squares) at the inflexion point, using experimental data from (**A**) and (**C**). Data are presented as mean ± s.d. of at least three independent experiments; complexes of rNP_113–200_-4D with H2A-H2B or crosslinked octamer are designated with orange and purple asterisks, respectively, while black asterisks mark protein aggregates.

Our working hypothesis is that the oligomeric structure of NP is necessary to stabilize complexes with histone octamers, whereas a single monomer is enough to bind a H2A-H2B dimer. To test this hypothesis, we characterized the interaction of the rNP_113–200_ peptide (C-terminal tail domain) and its phosphomimetic variant (rNP_113–200_-4D) (Fig. 2A) with nucleosomal histones. Both domains gave essentially the same results, thus only those obtained for the phosphomimetic peptide are shown. As seen by native electrophoresis, the tail domain formed a 1/1 complex with H2A-H2B dimers (Fig. 4A,D). Crosslinking of these complexes with BS^3^, a nonspecific crosslinker acting on primary amines that are closer than 11 Å, reinforced this interpretation (Fig. 4B). As expected, crosslinking of H2A-H2B yielded adducts corresponding to crosslinked histone dimers, tetramers and hexamers (Fig. 4B). The most abundant adduct observed for mixtures of the two proteins had an apparent molecular weight of around 38 kDa, which fits nicely with single tail domain/histone heterodimer. The susceptibility of H3-H4 to aggregate in the presence of the C-terminal domain of NP made analysis more difficult by either native electrophoresis or crosslinking (Fig. S6).

Our data demonstrate that none of the C-terminal domains by themselves could prevent dissociation of the histone octamer (Fig. 4C), in stark contrast with the natural, full-length pentameric chaperone (Fig. 2B). In the absence of the C-terminal domain, the octamer dissociated into its H2A-H2B and H3-H4 components, as Fig. 4C, lane 4, showed the same bands detected with isolated H2A-H2B or H3-H4 (lanes 1 and 3). Mixing either of the two histone types with the C-terminal domain of NP yielded aggregates with H3-H4 that are not resolved by electrophoresis (Fig. 4C, black asterisk; Fig. S6A) or a discrete complex with H2A-H2B (Fig. 4A,C, orange asterisk). The bands detected upon octamer addition to the C-terminal tail indicated complex formation with H2A-H2B dimers and aggregation with H3-H4, suggesting that this NP domain is not able to bind and stabilize the whole histone octamer. The octamer dissociated into its components that independently interacted with the C-terminal NP tail. In contrast, interaction of this domain with the crosslinked octamer rendered a complex in which 4 C-terminal tails formed a stable complex with the histone octamer (Fig. 4C, purple asterisk; Fig. 4D), indicating that several protomers must cooperate to stably bind it. This value also fits nicely with the aforementioned stronger binding of two C-terminal domains per pentamer with the base of the histone octamer. We cannot rule out that the core domain, besides ensuring the stability of the chaperone’s oligomeric structure, may establish additional interactions with the octamer that aid in stabilizing the complex.

**Figure 5:**
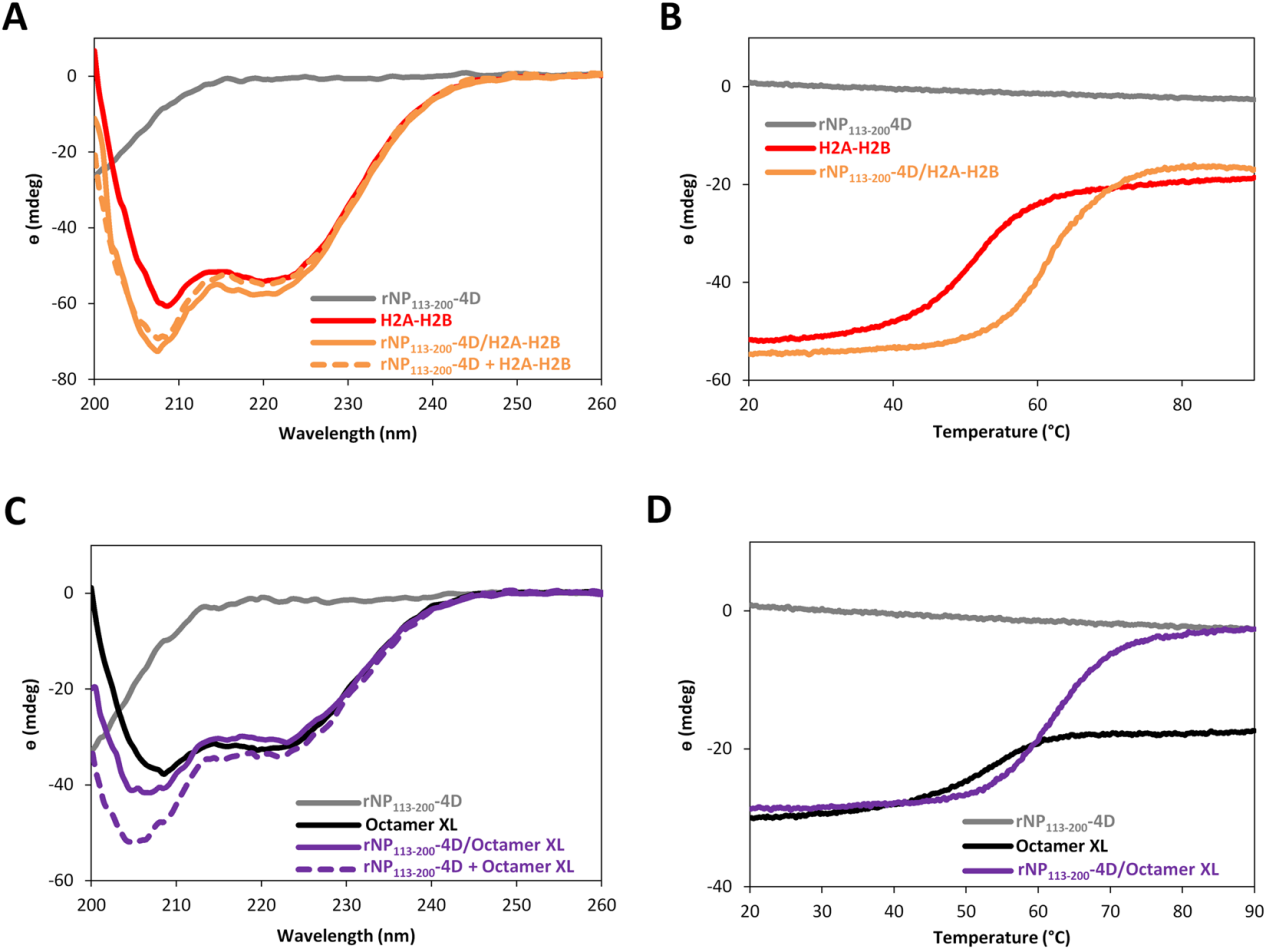
Interaction between the nucleoplasmin (NP) C-terminal domain and histones does not induce structural rearrangements in any of these proteins. Far-UV CD spectra of isolated rNP_113–200_-4D and H2A-H2B dimers (**A**) or crosslinked histone octamers (**C**), and of the complex between the chaperone domain and the histones. The spectra of the complexes were compared with the theoretical spectrum, obtained upon addition of the spectra of each protein component measured independently (dashed lines). The ellipticity of each protein component and of the complexes generated upon their mixing was monitored at 222 nm as a function of temperature (from 20 to 90 °C), to study the effect of rNP_113–200_-4D /H2A-H2B (**B**) and rNP_113–200_-4D/crosslinked octamer (**D**) complex formation on their thermal stability. For the sake of comparison, the same protein concentration (10 µM for rNP_113–200_-4D, 10 µM for H2A-H2B and 2 µM for the histone octamer) was used in all samples. Molar ellipticity could not be employed, as one of the samples is a protein mixture. The measurements were done in 10 mM K/K_2_PO_4_ pH 7.5, 150 mM NaF with a protein concentration of 10 µM for H2A-H2B and rNP_113–200_-4D and 2 µM for the histone octamer.

The intrinsically disordered, 80 residue-long, C-terminal domain of NP provides most of the interactions with all histone types. As NP forms distinct complexes with different histones, we wanted to know if this ability was mediated by structural rearrangements of the disordered chaperone domain upon binding of these ligands. We tested this possibility by analyzing the CD spectra of isolated histones and the NP C-terminal domain, and compared them with the spectrum of the corresponding mixture of the same proteins. The Far-UV CD spectrum of rNP_113–200_-4D showed a minimum at around 198 nm, which is representative of disordered proteins, thus supporting that either isolated or as part of the pentameric particle^30^, the C-terminal domain adopts an intrinsically disordered conformation (Fig. 5A). The minima at 208 and 222 nm in the spectrum of both H2A-H2B and the crosslinked octamer indicate the predominant helical fold of these proteins^31^. The ellipticity values at 222 nm obtained for the complexes between rNP_113–200_-4D and H2A-H2B dimers (1/1 molar ratio) or crosslinked histone octamer (5/1), were very similar to their theoretical ones, estimated by addition of the spectra of each independently measured protein component **(**Fig. 5A,C), ruling out extensive rearrangements of the major helical conformation upon association. As expected, complex formation resulted in stabilization, observed as a ~10 °C upward shift of the denaturation temperature (Fig. 5B,D).

### NP can transfer the complete octamer to DNA

**Figure 6:**
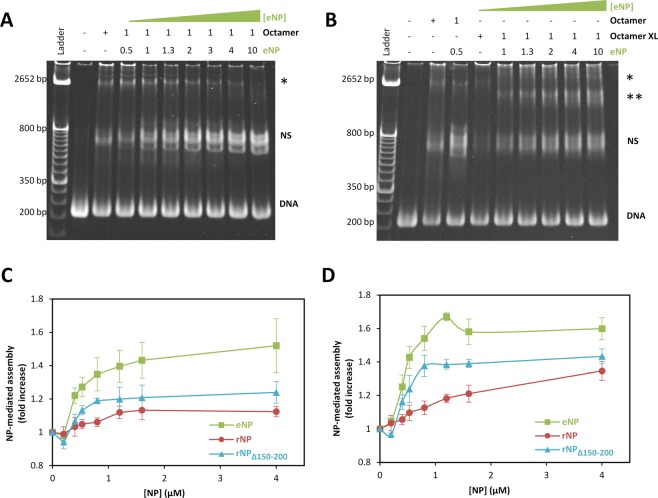
Nucleoplasmin (NP)-mediated transfer of histone octamers to DNA depends on chaperone post-translational modification and its C-terminal domain. 0.4 µM native (**A**) or crosslinked (**B**) histone octamers were incubated with increasing eNP concentrations for 1 h before the addition of 0.4 µM DNA. After 2 h at room temperature and 30 min at 42 °C, samples were analyzed by 6% native PAGE and stained with SRBR Green. The NP/Octamer molar ratio is noted above each lane. Bands marked with NS correspond to nucleosomes, and those with a single or double asterisk may be due to histone/DNA aggregates or to histone/DNA complexes generated upon transfer of crosslinked histone complexes. (**C,D**) NP-mediated nucleosome assembly was estimated as the intensity ratio of the nucleosome band in the presence of a given NP concentration and absence of the chaperone. Estimates are given for eNP (squares), rNP (circles; Fig. S6A,B) and rNP_Δ150–200_ (triangles; Fig. S6C,D), using native (**C**) or crosslinked (**D**) histone octamers. Lines connecting the symbols are just to guide the eye. Data are shown as mean ± s.d. of at least three independent experiments.

We next wanted to know whether NP could mediate transfer of histone octamers to DNA, facilitating nucleosome assembly. To this aim, NP/octamer complexes (1/0.5 molar ratio) made with eNP, rNP or rNP_Δ150–200_ were incubated with the plasmid pBlueScript II and the assembly of the nucleosomes was assessed by the micrococcal nuclease digestion method (Fig. S7A). The results showed that the three NP variants mediated the assembly of nucleosomes although we could not obtain quantitative efficiency differences due to experimental limitations. Thus, we resorted to electrophoretic mobility shift assays (EMSA) with a 207 bp linear DNA to estimate the transfer efficiency of the different NP species. Protein complexes obtained at different chaperone/octamer molar ratios were incubated with a constant concentration of 207 bp linear DNA (Figs 6A and S7C,E). The resulting bands were assigned to free DNA and histone octamer/DNA complexes (nucleosomes), as compared to control nucleosomes obtained by the salt dialysis method (Fig. S7B). It is worth mentioning that NP does not interact with chromatin or nucleosomes in these experimental conditions^7^. Our data showed that although spontaneous nucleosome assembly occurred, all NP variants enhanced nucleosome formation, suggesting that they could mediate transfer of histone octamers to DNA, albeit with significantly different efficiencies (Fig. 6C). The greatest efficiency corresponded to phosphorylated eNP, which transferred histones to DNA significantly better than rNP_Δ150–200_ and unmodified rNP. This behavior paralleled reasonably well the ability of these proteins to form the high molecular mass complex with histone octamers. Octamer deposition onto DNA could be either sequential (if core histones are transferred as separate components) or alternatively the octamer could be deposited as a unit. As both alternatives would give rise to nucleosome assembly, one way to experimentally determine if the latter occurs was to follow the NP-mediated transfer of crosslinked octamers (Figs 6B and S6B,D). The data showed that the three NP variants could transfer crosslinked octamers to DNA, with relative transfer efficiencies similar to those observed for the natural octamer (Fig. 6D). Interestingly, although rNP formed a stable complex of higher apparent molecular weight with crosslinked octamers, DNA deposition by this complex was not as efficient as that found for eNP. According to the widely used definition of histone chaperones as proteins that bind histones and stimulate their transfer to DNA^1,32^, these data indicate that NP is able to enhance transfer of the complete octamer to DNA, and that the same NP features that ensure chaperone/octamer stability, namely PTMs and exposure of the polyGlu tract, facilitate the transfer process.

## Discussion

NP is a histone chaperone that binds H2A-H2B and H3-H4 dimers and tetramers with high affinity. In this work, we explored the ability of this chaperone to interact with histone octamers and transfer them to DNA, a characteristic that is uniquely found in NP among all the histone chaperones. The global structure of the eNP/octamer complex was previously analyzed by electron microscopy using negative staining, revealing an ellipsoidal particle made of two chaperone pentamers embracing the octamer^16^. Two alternative binding models have been previously proposed. The first is based on the crystal structure of the core domain of NP^12–14^, and proposes that a decamer interacts with the octamer through its lateral face. This model is further supported by FRET experiments using recombinant chaperones^14^, which can also be explained with the structure presented herein. Our data indicate that the recombinant protein has a tendency to self-associate forming decamers, and that this behavior is not observed for natural eNP (Figs 2B and S3). The second model suggests that a single recombinant NP pentamer can bind two H2A-H2B dimers and a H3-H4 tetramer (a histone octamer equivalent) under physiological ionic strength, without adopting an octamer structure^24^. In our hands, the interaction of rNP with non-crosslinked histone octamers is rather unstable. In the current study, our data clearly showed that eNP can both bind to preformed histone octamers, and interact with their components to mediate their assembly. The 3D reconstruction of this complex showed that the distal face of two NP pentamers interact on opposite sides of the octamer, embracing its structure and giving rise to an elongated and curved complex. Complexes other than the one described here might occur with the recombinant protein but not with natural eNP. In the structure presented herein, the interaction between the NP pentamers and histone octamer is loose, and the tails of the chaperone can slide, making the NP/octamer complex a very heterogeneous one that prevented its determination at high resolution. The interaction between the four tails of the NP pentamer and the rectangular prism structure of the octamer was shown to be very asymmetric, with two tails per NP pentamer concentrated at the base of the histone octamer, where the two H2A-H2B dimers are located and where the octamer is assembled. This asymmetry in the interaction is very likely favored by the electrostatic attraction between the highly negative nature of the NP tails and the positive charges concentrated at the base of the octamer. This interaction explains why this region of the octamer is clearly more protected when the NP/octamer complex is subjected to proteolysis^16^. It also agrees with an interaction between the A2 tract of the tail domain with the N-terminal region of the H2A histone fold and the C-terminal region of H2B, recently found by NMR^28^. The interpretation, in light of the EM results, that the four nucleosomal histones bound to eNP adopt a compact octameric conformation, is further supported by FRET data^16^, showing a similar energy transfer efficiency between H2B and H4 when the octamer components are bound to eNP under experimental conditions that favor octamer dissociation, e. g., 240 mM NaCl, or in 2 M NaCl, conditions that stabilize the octamer in a structure very similar to that found in the nucleosome^29^.

PTMs, mainly phosphorylation, of the C-terminal tail induces a conformational transition in the tail structure from a compact state, in which the acidic polyGlu tract electrostatically interacts with the basic NLS located at the C-terminus of the same monomer, to a more open conformation that exposes both charged tracts^28^. Hence, PTM of NP not only increases the negatively charge density at the distal face of the chaperone, but also promotes exposure of the acidic polyGlu tract. Thus, the rearrangements that the NP C-terminal domain undergoes after PTM favors electrostatic interaction with the basic ligand^17,20^. Additional interactions between H3-H4 and the core of the chaperone may further stabilize the histone octamer structure.

One of the most striking features of histone chaperones is their different oligomeric architectures^2,33^, which might be related to the ligand they interact with in order to fulfill their biological activity. One reason that could explain formation of oligomeric structures such as the NP pentamer is the need to bind large histone oligomers. The different oligomeric structures of histone chaperones could aim for stabilization of the distinct oligomerization states of histones. The pentameric structure of NP would ensure the proper size of the binding region to interact with the octamer, and the number of tail domains necessary to stabilize the complex. This interpretation is supported by analysis of the interaction of histone chaperones with the different histone structures. Monomeric chaperones can bind H2A-H2B or H3-H4 dimers^34–36^, whereas complex formation with H3-H4 tetramers requires dimeric chaperones^33,37–39^. Another example of oligomeric chaperones is Vps75, which can interact with a H3-H4 dimer or tetramer either as a pair of dimers or as a tetramer, depending on histone concentration, and thus could potentially assemble new H3-H4 tetramers or stabilize those evicted from chromatin^40^. Human FACT (hFACT) is a heterodimeric complex that can bind simultaneously to a H2A-H2B dimer and one H3-H4 tetramer, because the basic ligands interact with different regions of the histone chaperone, in turn also mediating their interaction^41,42^. Histone octamer binding could impose an additional requirement for the oligomerization state of histone chaperones due to the larger size of the ligand. The coordinated action of several protomers of two pentameric NPs would provide the interacting surface to “cage” the octamer. Our data demonstrate that a single NP tail domain can form stable 1/1 complexes with histone H2A-H2B dimers. They agree with our previous EM single-particle reconstruction of the NP/H2A-H2B complex, which shows a star-like structure with one histone dimer interacting with one C-terminal tail at the distal face of the eNP pentamer^15^, and a recent NMR study^28^. This stoichiometry has also been confirmed by ITC, SAXS, fluorescence, and analytical ultracentrifugation analyses of NP/H2A-H2B complexes^15,21,22^. In stark contrast, a crosslinked octamer binds 4 tail domains, pointing to the cooperation between several NP protomers to stabilize the complex with the histone octamer. The need of two pentamers to form a stable complex with the octamer is most likely due to the length of the flexible NP arms that cannot wrap around the entire octamer structure unless two chaperone molecules cooperate to surround the basic ligand. The similarity of the structures of the octamer bound to NP and in the nucleosome would promote transfer of histone octamers from NP to DNA, avoiding the energetic penalty of a significant conformational rearrangement.

The intrinsically disordered nature of the C-terminal tails of the chaperone could also be involved in the adaptation of the binding surface on the distal face of the chaperone pentamer to histone dimers, tetramers, and, as shown here, octamers. Intrinsically disordered regions are highly dynamic because they lack stable secondary structure, and sample an ensemble of different conformations. Upon ligand binding, they can either fold into different conformations depending on the ligand or remain disordered. Our data suggests that the tail domain of NP remains mainly unordered in complex with H2A/H2B or histone octamers, and therefore that interaction with these basic ligands does not induce a characteristic fold in this domain, as recently found for the interaction of H2A/H2B with a similar tail fragment^28^.

The compact nature of chromatin inhibits processes that occur on DNA, such as replication, repair and transcription^43–45^. To modulate these processes, the basal transcription machinery is assisted by numerous factors that increase the plasticity of nucleosomal DNA by covalently modifying histones, incorporating histone variants, and remodeling chromatin through ATP-dependent and independent processes^46,47^ and references therein. Transcription elongation causes displacement of the core histones in a manner that depends on the transcription rate^45,48^. On moderately active genes, H2A-H2B histones are displaced/exchanged at a much higher rate than H3-H4 histones^48–50^. The situation is however different during intense transcription, as all core histones are displaced/exchanged at the transcribed regions^48,50^, thus implying that nucleosome disruption and reassembly during transcription must be very efficient. Histone chaperones are involved in both chromatin disassembly and reassembly, both activities being essential in transcription-associated histone exchange. RNA polymerase II may provide the force necessary to displace the nucleosome, which can then be chaperoned and reloaded by histone chaperones such as NP. A chaperone capable of interacting with or assembling the histone octamer would mediate its fast redeposition onto DNA during intense transcription. Although extrapolation of our data to what might occur *in vivo* is not straightforward, the above interpretation is supported by the fact that NP has been associated with active chromatin and transcription products, and it is hardly detected in transcriptionally inactive cells^51–53^.

**Figure 7:**
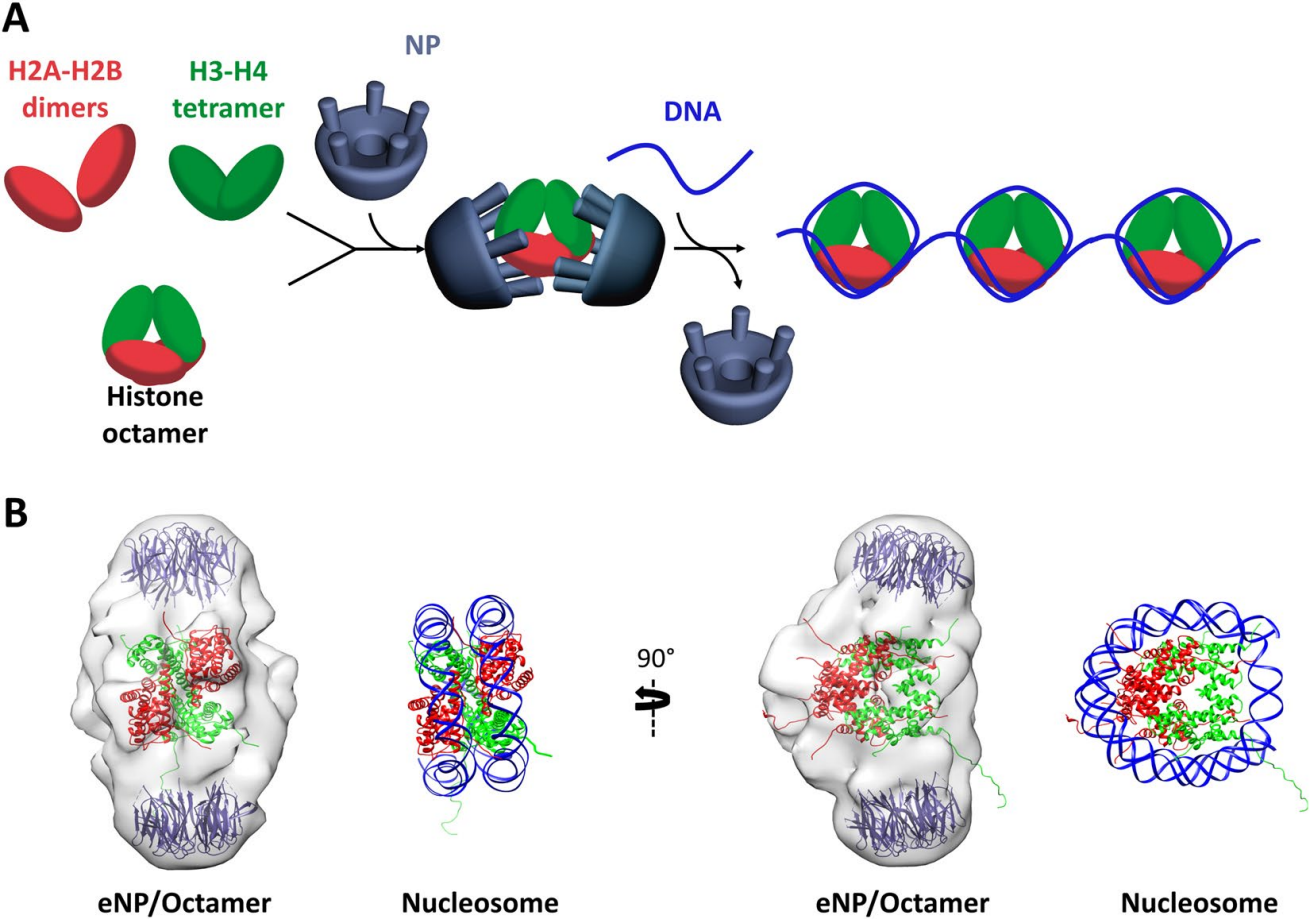
Model for nucleoplasmin (NP)/histone octamer complex formation and chaperone-mediated octamer transfer to DNA. (**A**) NP can either bind sequentially the histone octamer components, assembling an octamer-like particle, or interact with preformed, stable histone octamers. The complex involves interaction of two NP pentamers and a histone octamer. In the presence of DNA, the NP-bound octamer is transferred to DNA, leading to nucleosome formation. (**B**) Comparison of the octamer structure (pdb 1AOI) docked into the CryoEM structure of the eNP/octamer complex and in the nucleosome. Note that the DNA strands are located in the same regions of the chaperone tails. The histone octamer employs common interacting surfaces to bind DNA or NP, offering a rationale for the competition between NP and DNA for octamer binding.

The model of NP/histone octamer interaction that can be drawn from our data is summarized in Fig. 7A. NP can either bind sequentially the components of the histone octamer or interact with preformed octamers by using several protomers of two chaperone pentamers. The NP-bound octamer adopts a nucleosome-like conformation and employs common interacting surfaces to bind DNA or NP. This is clearly observed in Fig. 7B, which shows the structure of the octamer docked into the NP/histone octamer complex and in the nucleosome, indicating that the DNA molecule and the tail domains of the two NP pentamers that embrace the octamer occupy the same volume around the basic ligand. This would ensure that they compete for octamer binding, thus favoring transfer of NP-bound octamers to DNA for nucleosome assembly.

## Materials and Methods

### Protein purification

Egg NP (eNP) from *X. laevis*, recombinant NP (rNP) (Genbank accession X04766.1), and truncated variants lacking either the last 50 (rNP_Δ150–200_) or 80 residues (rNP_Δ120–200_) were expressed and purified as described^30^. A sequence encoding the C-terminal domain of NP (rNP_113–200_) was cloned into a pET28a plasmid and its phosphomimetic mutant (rNP_113–200_-4D) was obtained by mutating serine residues 148, 177, 178 and 181 to aspartate using the *QuickChange II Site-Directed Mutagenesis kit* (Agilent Technologies). Both wild-type and phosphomimetic tail domains were purified using the same procedure. Briefly, expression in *E. coli* BL21 (DE3) was induced at 37 °C with 1 mM isopropyl-β-D-thiogalactoside (IPTG) when the OD_600_ reached 0.6–0.8 and cells were harvested after 2 h. The bacterial pellet was resuspended in buffer A (350 mM NaCl, 10 mM β-mercaptoethanol, 10 mM imidazole, 1 mM phenylmethylsulfonyl fluoride (PMSF), 5% glycerol (w/v), 20 mM Tris-HCl pH 8) and cells were disrupted by sonication. The soluble fraction was separated by ultracentrifugation and the supernatant was loaded onto a 5 ml *HisTrap FF* affinity column (GE Healthcare) previously equilibrated in buffer A. After elution with buffer B (buffer A plus 400 mM imidazole), the sample was dialyzed against buffer A. His-tags were cleaved with 0.5 U thrombin/mg protein (*Thrombin Restriction Grade*, Novagen) for 2 h at room temperature (RT) and the reaction was stopped with 1 mM PMSF. Thrombin-treated sample was loaded again onto a 5 ml *HisTrap FF* affinity column, dialyzed against buffer C (100 mM NaCl, 20 mM Tris-HCl pH 7.5) and loaded onto a *Q-Sepharose* column equilibrated with the same buffer. The protein was eluted with a linear gradient (0.1 to 0.8 M NaCl) and stored at −20 °C in buffer D (150 mM NaCl, 5% glycerol [w/v], 20 mM Tris-HCl pH 7.5). NP concentration was determined by bicinchoninic acid assay (Sigma), and is provided for its pentameric form, except in the case of the C-terminal domain mutant rNP_113–200_-4D.

Natural-source H2A-H2B, H3-H4 and histone octamers were obtained from chicken erythrocyte chromatin upon elution from a hydroxyapatite column^54^ and kept at 4 °C in 2 M NaCl until use. Recombinant histones were expressed, purified and reconstituted as described^55,56^. The concentration of natural histones was determined by absorbance at 230 nm, using extinction coefficient values (ε_230_) of 4.35 cm^2^ mg^−1^ and 4.10 cm^2^ mg^−1^ for the dimeric forms of H2A-H2B^15^ and H3-H4, respectively^16^, and 4.20 cm^2^ mg^−1^ for the histone octamer^24^, all in water. The concentration of recombinant histones and crosslinked histone octamer was estimated by densitometry using known amounts of H3-H4, H2A-H2B or histone octamers as standards. Histone concentration is given for the dimeric species of H3-H4 and H2A-H2B. Molar ratios are expressed as NP pentamer/H3-H4 or H2A-H2B dimer or histone octamer, except for the NP C-terminal proteins, which is given for the monomer.

### Crosslinking

Histone octamers were crosslinked with dimethyl suberimidate (DMS) as previously described^57^. Briefly, histone octamers were incubated for 1 h at RT in buffer containing 2 M NaCl and 0.2 mM EDTA, 0.1 M sodium-borate pH 10, with DMS and protein at a final concentration of 0.4 mg/ml and 1.5 mg/ml, respectively. Crosslinking was stopped by adding 0.25 M Tris-HCl pH 7.6 to a final concentration of 50 mM. Samples were analyzed by 18% SDS-PAGE and the estimated crosslinking efficiency was 75%, as quantified by densitometry using a GS-800 Calibrated densitometer and the Quantity One 4.5.0 program (Bio-Rad).

Complexes between the NP tail domain and histones were crosslinked using bis(sulfosuccinimidyl)suberate (BS^3^). Histones and NP were mixed at the corresponding final concentrations in buffer containing 150 M NaCl, 10 mM Hepes pH 7.5 for 1 h at RT. BS^3^ was added to the protein mixtures (0.25 mM final concentration), and samples were incubated at RT for 15 min. Reactions were quenched by adding 1 M Tris-HCl pH 7.5 to a final concentration of 50 mM. Samples were analyzed by NuPAGE Novex 4–12% Bis-Tris Gels (Invitrogen) in MES buffer.

### Mobility shift assays of NP/histone complexes

NP/octamer complexes were characterized with the NativePAGE™ Novex™ 4–16% Bis-Tris Protein Gels (Invitrogen). NP (2 µM) was mixed with different histone concentrations and incubated in 150 mM NaCl, 25 mM Tris-HCl, pH 7.6 at RT for 1 h. Proteins were stained with Coomassie Brilliant Blue and the intensity of the bands was estimated by densitometry. The 440 kDa complex fraction was calculated as the intensity of the 440 kDa band relative to that of NP in the absence of histones. The contribution of histones to the intensity of the complex band could not be considered in the estimation, as they did not enter the gel under these experimental conditions, explaining maximum values greater than 1. It should be noted that all experiments were carried out with native and crosslinked octamers. The use of the crosslinked basic ligand pursued to demonstrate that NP can also bind a stable octamer structure.

Interaction between histones and the NP C-terminal domain under native conditions was analyzed by electrophoretic mobility shift of histones upon chaperone binding, as previously described^58^, with the following modifications. Samples containing a fixed amount of histones (10 μM for dimers H2A-H2B and H3-H4, and 2.5 μM for the histone octamer) and increasing rNP_113–200_-4D concentrations were incubated at RT for 1 h in buffer containing 150 mM NaCl and 20 mM Tris-HCl pH 7.6. Afterwards, they were analyzed by 8% native PAGE in buffer containing 1.5 mM EDTA, 20 mM sodium acetate and 40 mM Tris-HCl pH 7.2, running the samples for 3.5 h at 100 V from the positive to the negative electrode, and stained with Coomasie Brilliant Blue.

### Nucleosome assembly

Salt dialysis and NP-mediated assembly of nucleosomes was carried out using a 207 base pair DNA, comprising the 5S rRNA gen of *Lytechinus variegatus*, as previously described^22^. The efficiency of the process was estimated by the factor (X_i_/X_0_); where X_i_ is the intensity of the nucleosome band in the presence of NP, and X_0_ that measured in the absence of the chaperone. NP-mediated assembly of nucleosomes was also assessed by a micrococcal nuclease digestion assay as described in Supplementary Methods.

### Circular dichroism (CD)

CD spectra were recorded at 25 °C on a Jasco J-810 circular dichroism spectropolarimeter, using rectangular quartz cuvettes with 1 mm path length in buffer containing 150 mM NaF and 10 mM K_2_H/KH_2_PO_4_ pH 7.5. Protein concentration was 10 µM for rNP_113–200_-4D, 10 µM for H2A-H2B and 2 µM for the histone octamer. Each spectrum represents the average of 15 scans, collected from 200–260 nm, with spectral bandwidth of 1 nm and a response time of 1 s. Thermal stability of the samples was estimated by measuring the temperature dependence of the ellipticity value at 222 nm during heating at 60 °C/h.

### Fluorescence spectroscopy assays

H2BT112C was labeled with Alexa 488 (Invitrogen) at a protein/fluorescent probe molar ratio of 1/5, in 1 mM Tris(2-carboxyethyl)phosphine (TCEP), 6 M guanidine hydrochloride, 20 mM Tris-HCl pH 7.2, and incubated overnight at 4 °C as indicated^59^. Labeled histones were mixed with H2A, H3C110A and H4 T71C to reconstitute histone octamers^55^, which were purified by size exclusion chromatography with a Superdex 200 16/60 column (GE Healthcare). The concentration of labeled histone octamers was estimated by densitometry using known amounts of standards.

Fluorescence at 519 nm was measured in a Horiba Jobin Yvon Fluoromax-3 Spectrofluorometer, after excitation at 495 nm, using excitation and emission slits of 8 nm. The affinity of each NP species for the histone octamer was estimated by titrating 5 nM of labeled histones with increasing chaperone concentrations. Experiments were performed in 150 mM NaCl, 1 mM DTT, 0.1 mg/ml BSA and 20 mM Tris-HCl, pH 7.5. Data were analyzed using a ligand-depleted model, which considers that two eNP pentamers bind a histone octamer, as described in^22^ for the interaction of H3-H4 with NP (Supplementary Information).

### Electron microscopy (EM) and image processing

The eNP/histone octamer (1/0.5 molar ratio) complex was prepared by incubating NP and native histone octamers in 240 mM NaCl, 2 mM MgCl_2_ and 25 mM Hepes pH 7.5, during 1 h at room temperature. Afterwards, complexes were crosslinked for EM by the gradient fixation (GraFix) technique as described in^60^, with minor changes. The gradient (10–30% glycerol) was prepared in the above buffer, using a Gradient Master 107 (BIOCOMP, Fredericton, NB, Canada); the 30% glycerol solution contained 0.15% glutaraldehyde. The sample loaded in the glycerol fixation gradient was spun on a TST 41.4 swinging bucket rotor (Kontron) at 30,000 rpm for 16 h at 4 °C and fractionated after centrifugation. Complexes were identified by 12.5% SDS-PAGE and 4–16% native PAGE analysis of the fractions. Crosslinking was used to reduce the flexibility/ heterogeneity of the complex.

For cryoEM sample preparation, Quantifoil grids (R1.2/R1.3 300 mesh grids; ref. Q09684) were covered with a thin carbon layer (4 nm) and glow-discharged for 20 s. Aliquots (5 µl) of the NP/octamer complex were incubated with the grid (2–5 min), blotted and plunged into a liquid ethane chamber. All operations were performed on a Leica CPC manual plunger. Images were acquired in a FEI Titan Krios electron microscope at 300 kV (Laboratory of Molecular Biology, Cambridge). A Falcon II detector was used at a calibrated magnification of 80,500 (1.74 Å/px simple resolution), and a dose rate of ~2.5 electrons/Å^2^. Exposures of 1.5 s were fractionated into 34 movie frames.

Movie correction was carried out using MOTIONCORR^61^, CTF correction with CTFFIND4^62^ and SCIPION, a suite of programs, was used for all the image processing steps^63^. A total of 166,976 particles were selected manually from 897 micrographs, and after 2D classification, 11,438 particles were used for the final 3D reconstruction. Resolution of the final 3D model (17.4 Å) was estimated based on the Fourier shell correlation^64^ criterion. Visualization of the 3D models and docking of the atomic structures into EM volumes was performed manually using USCF Chimera^65^.

### Accession numbers

The three-dimensional EM map has been deposited in the EM Data Base (http://www.ebi.ac.uk/pdbe/emdb/) with accession number EMD-0323.

## Supplementary information

Estructural Insights into the ability of nucleoplasmin to assemble and chaperone histone octamers for DNA deposition
