# Supplementary material for: Structural insights into the ability of nucleoplasmin to assemble and chaperone histone octamers for DNA deposition
Source: Sci Rep. 2019 Jul 1;9:9487. doi: 10.1038/s41598-019-45726-7 (PMC6602930; doi:10.1038/s41598-019-45726-7)
Supplement: Supplementary file 1 — Estructural Insights into the ability of nucleoplasmin to assemble and chaperone histone octamers for DNA deposition [file 41598_2019_45726_MOESM1_ESM.pdf]

Supplementary Information

Structural insights into the ability of nucleoplasmin to assemble and chaperone histone octamers for DNA deposition

Aitor Franco, Rocío Arranz, Noelia Fernández-Rivero, Adrián Velázquez-Campoy, Jaime Martín-Benito, Joan Segura, Adelina Prado, José M. Valpuesta and Arturo Muga

SUPPLEMENTARY MATERIALS AND METHODS

Data analysis of fluorescence spectroscopy assays

In the analysis of the fluorescence titrations it was assumed that the fluorescence signal was only due to the histone octamer, which changed upon nucleoplasmin binding. A ligand depletion model (that is, the free reactant concentrations for octamer and nucleoplasmin were not approximated by their total concentrations) was used in which two binding (independent and identical) sites for nucleoplasmin on the histone octamer have been considered, as described in [1] for the interaction of H3-H4 with nucleoplasmin. Mass conservation for nucleoplasmin coupled to chemical equilibrium leads to the following equation:

Eq. 1:

$$[L]_T = [L] + [PL] = [L] + [P]_T \frac{2K[L]}{1 + K[L]}$$

where $[P]_T$ and $[L]_T$ are the total concentrations of histone octamer (considered the macromolecule) and nucleoplasmin (considered the ligand), respectively, $[L]$ is the free concentration of nucleoplasmin, $[PL]$ is the concentration of octamer-nucleoplasmin complex, and K is the association constant for the nucleoplasmin/octamer interaction (being the dissociation constant K_d its inverse). At each experimental point in the fluorescence titration the total

concentrations $[P]_T$ and $[L]_T$ are known. Solving this quadratic equation for $[L]$, assuming a value for the association constant K, allows calculating of the concentration of octamer-nucleoplasmin complex, $[PL]$, and the measured fluorescence intensity signal, F, as a function of the reactant concentrations (in particular, the total concentration of nucleoplasmin, $[L]_T$) at each experimental point along the titration:

Eq. 2:

$$F = F_0 + \Delta\varepsilon[PL] = F_0 + \Delta\varepsilon[P]_T \frac{2K[L]}{1 + K[L]}$$

where F_0 is the initial fluorescence value of the octamer solution (in the absence of nucleoplasmin), and $\Delta\varepsilon$ is the change in fluorescence emission coefficient for the octamer as it gets saturated with nucleoplasmin. The binding isotherm was constructed by plotting the measured fluorescence intensity signal as a function of the nucleoplasmin concentration. Non-linear least squares regression data analysis was performed implementing this model in Origin 7 (OriginLab) in order to estimate F_0, $\Delta\varepsilon$, and K. There was no need to consider neither two non-identical binding (with different dissociation constants) sites nor two cooperative binding sites (through an effective Hill coefficient in the previous equations) for nucleoplasmin in the histone octamer.

Micrococcal nuclease digestion assay

NP/octamers complexes (1/0.5 molar ratio) were incubated for 1 h at room temperature in buffer 2 mM $MgCl_2$, 150 mM NaCl, 20 mM Tris-HCl pH 7.5. Complexes were added to pBlueScript II plasmid in 2 mM $MgCl_2$, 150 mM NaCl, 0.2 mM EDTA, 20 mM Tris-HCl pH 7.5 at a plasmid/octamer (1/0.8 μg/μg) ratio and incubated 2 h at room temperature. As a control, the same experiment was done in the absence of NP. The micrococcal nuclease digestion assay was carried out essentially as previously described [2]. After addition of 1 mM $CaCl_2$ (final concentration), each reaction mixture was digested different times with 0.5 U micrococcal nuclease (Sigma), and with 25 μg proteinase K for 1 h at 37º C. Purified DNA was run in 1.5% agarose native gel at 70 V in TAE (1 mM EDTA, 1% (v/v) glacial acetic acid, 40 mM Tris-HCl pH 8.0) buffer and stained with ethidium bromide.

Two-dimensional electrophoresis

To analyze the protein composition of the 440kDa molecular weight band we carried out a 2D electrophoresis analysis. eNP (2 μM) was initially mixed with H2A-H2B or H3-H4, at a 1/1 pentamer/dimer ratio, in buffer 25 mM Tris-HCl pH 7.5, 240 mM NaCl, 2 mM $MgCl_2$. After a 30 min incubation, the other histone, e.g., H3-H4 or H2A-H2B, respectively, was added to the mixture. The resulting eNP/H2A-H2B/H3-H4 complexes (1/0.5 pentamer/octamer molar ratio) were loaded in a native 4-16% PAGE precast Novex Native Bis-Tris Gel (Life Technologies) and the unstained gel strips were cut, treated as described by the manufacturer, analyzed by 12.5 % SDS-PAGE and stained with Coomassie Brilliant Blue.

SUPPLEMENTARY FIGURES

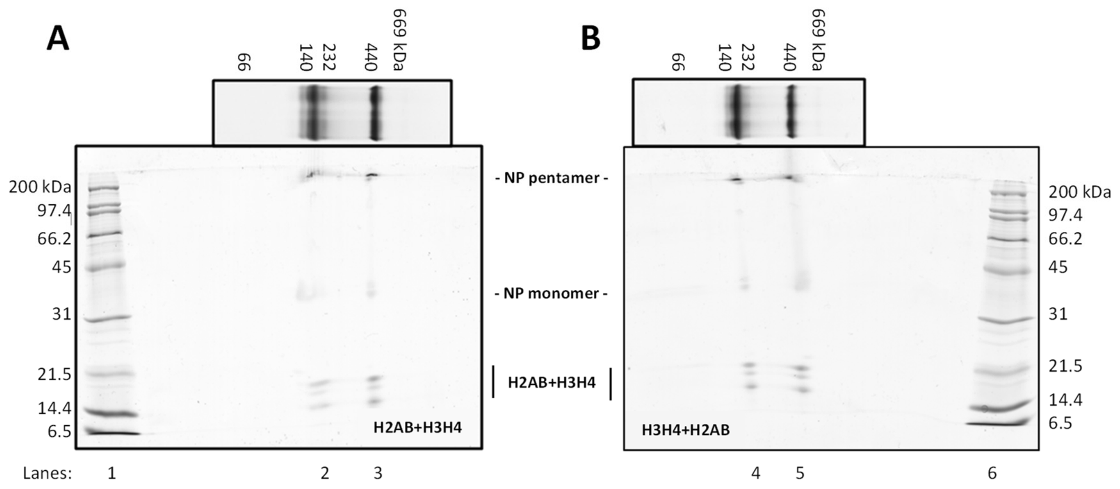

Figure S1. Two-dimensional electrophoresis of the eNP/H2A-H2B/H3-H4 complexes. The samples obtained after mixing eNP/H2A-H2B and eNP/H3-H4 (1/1) complexes with equimolar amounts of H3-H4 **(A)** and H2A-H2B **(B)** dimers, respectively, were analyzed by 2D electrophoresis. First dimension, native Bis-Tris 4-16% PAGE, and second dimension 12.5% SDS-PAGE. The upper boxes show the bands corresponding to the low and high molecular weight complexes in Native PAGE gels stained with Coomassie-Blue. The components of these complexes were resolved by SDS-PAGE (lower gels). Lanes 1,6, MW markers; 2,4, low molecular weight band components; 3,5, high molecular weight band components. The position of the core histones, NP monomer and NP pentamer are indicated. The low molecular weight bands contain free and NP/histone dimer complexes, which are not completely resolved, while the high molecular weight one corresponds to the NP/octamer complex, as previously described [2].

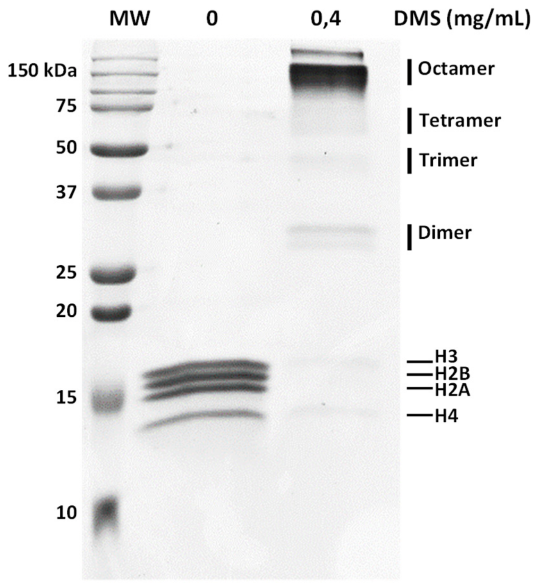

Figure S2. Crosslinking of the histone octamer. The histone octamer was crosslinked using DMS and analyzed by 18% SDS-PAGE. As a control, the histone octamer without crosslinker was also analyzed. 75% of the protein was crosslinked as an octamer, the remaining protein being present as dimers, trimers, tetramers and higher oligomerization states. Lane MW, molecular weight markers in kDa.

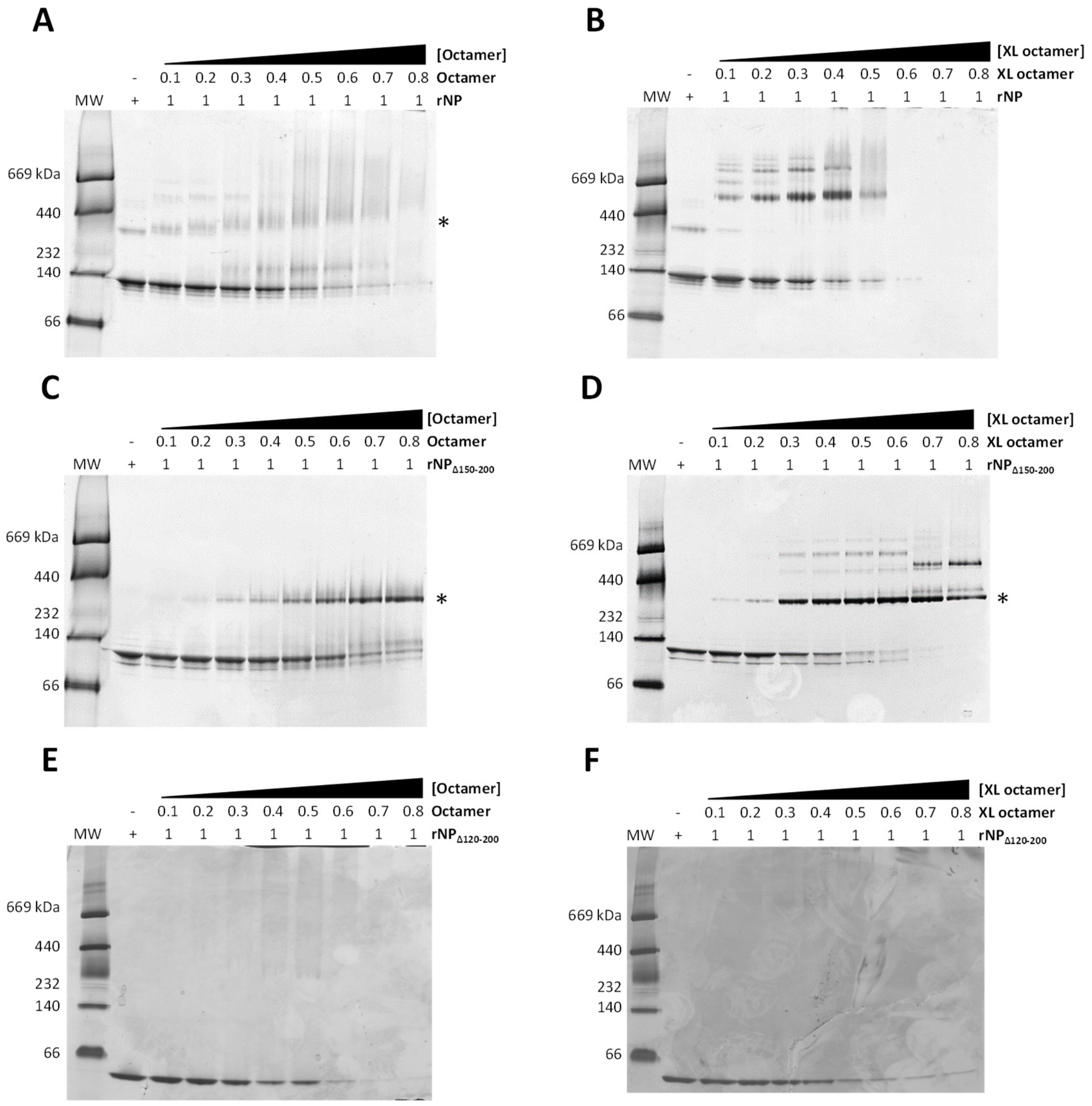

Figure S3. Phosphorylation and exposure of the acidic tract of nucleoplasmin (NP) regulates the interaction of NP with histone octamers. Titrations of rNP **(A, B)**, rNP$_{\Delta 150-200}$ (**C, D)** and rNP$_{\Delta 120-200}$ **(E, F)** with different amounts of native **(A, C, E)** or crosslinked **(B, D, F)** histone octamers. Samples containing 2 µM of the different NP variants and the concentrations of histone octamer to achieve the corresponding NP/histone molar ratios were analyzed by 4-16% native PAGE gels stained with Coomassie-Blue. The complex formed by two NP pentamers and a histone octamer is marked with an asterisk.

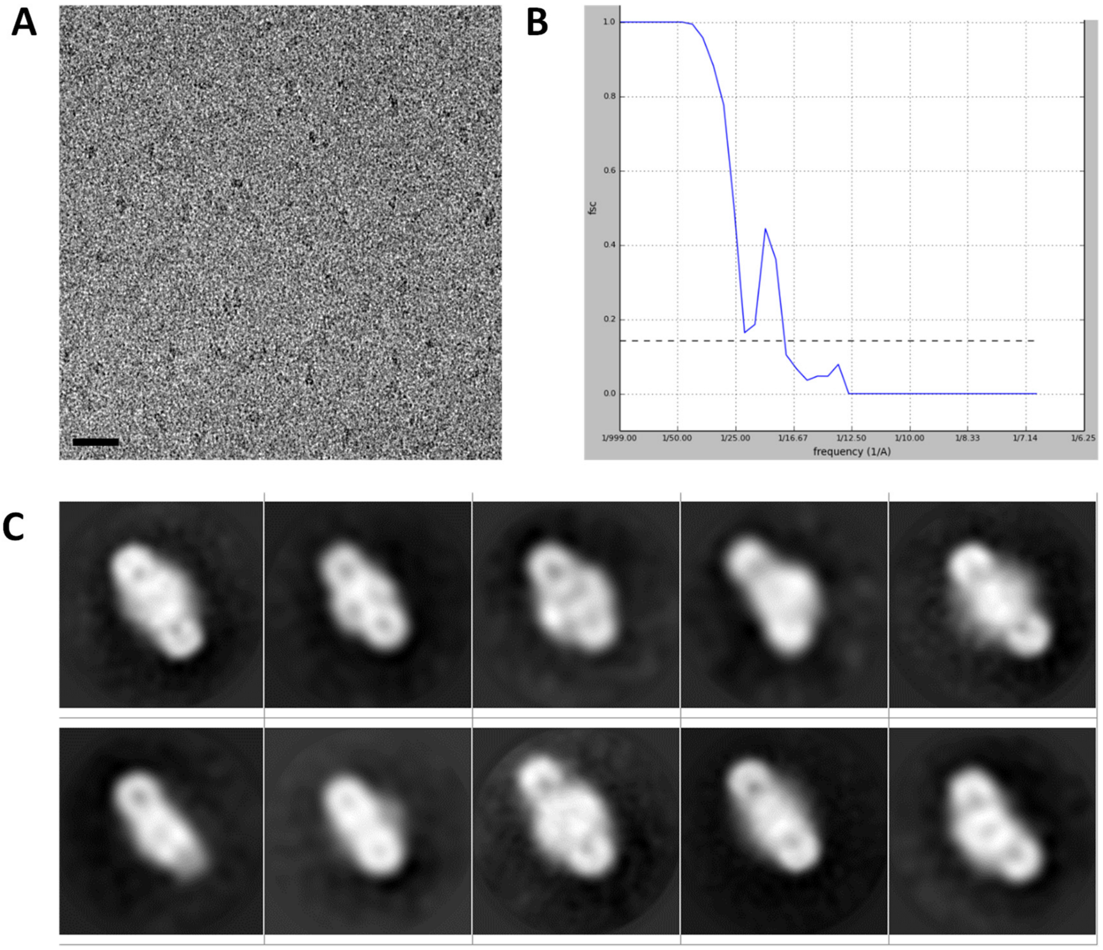

Figure S4. 3D reconstruction of the NP/octamer complex. (A) Cryoelectron microscopy image of the NP/octamer complex. Bar indicates 500 Å. **(B)** Plot of the Fourier Shell Correlation coefficient vs. resolution between two independent reconstructions of the NP/octamer complex using the gold-standard method. The resolution obtained for a signal to noise ratio (SNR) of 0.143 is 17.4 Å. **(C)** Reference-free 2D classification of the nucleoplasmin (NP)/octamer complex. The classification was performed using Relion 2.0 and shows the size variability of the complex, in particular its length. This high variability prevented structural determination of the complex at a high resolution.

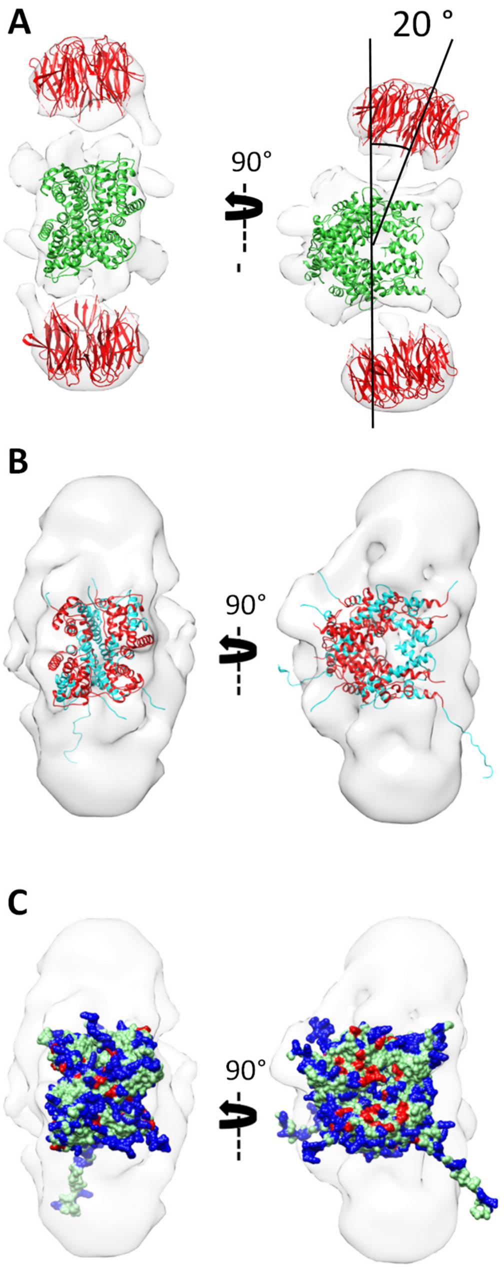

Figure S5. Comparison of the histone octamer structure in complex with nucleoplasmin (NP) and in the nucleosome. (A) Two orthogonal views of the NP/octamer complex with docking of two molecules of the atomic structure of the NP core (pdb 1K5J) and of the histone octamer (pdb 1AOI), as in Fig. 3B. The threshold of 3D reconstruction has been increased to 4σ to show the regions with the highest density, which correspond to the areas where these three molecules are located. **(B)** The same two orthogonal views with the docking of the atomic structure of the histone octamer, colored red in regions of the histone octamer that are protected from proteolysis by the two NP pentamers, and colored light blue in areas proteolyzed in the presence of the chaperone [3]. **(C)** The same views with docking of the histone octamer, with surface representation of the histone octamer showing positive (blue) and negative residues (red). Note that the positively charged residues are concentrated in the area where interaction with the NP tails is stronger (front side of the left image) whereas the negative residues are mostly located outside this area.

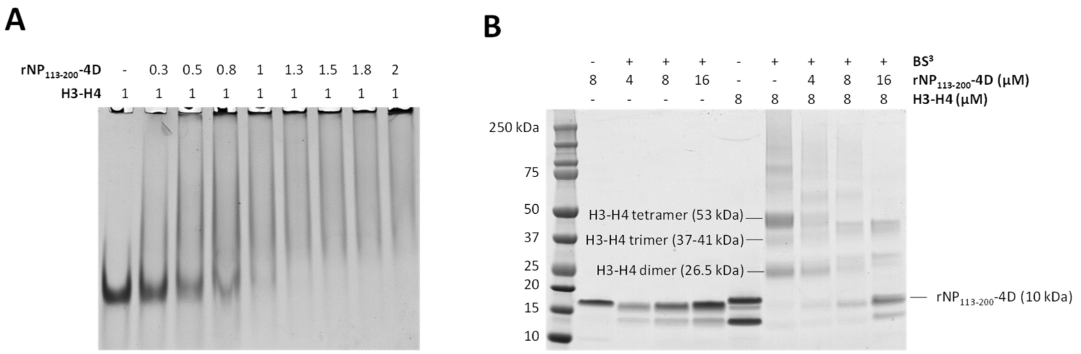

Figure S6. The nucleoplasmin (NP) C-terminal domain is not able to stabilize H3-H4. (A) H3-H4 was incubated with increasing amounts of $rNP_{113-200}$-4D for 1 h, and samples were analyzed by 8% native PAGE. **(B)** H3-H4/$rNP_{113-200}$-4D complexes were crosslinked with bis(sulfosuccinimidyl)suberate (BS^3) and analyzed by denaturing NuPAGE Novex 4-12% Bis-Tris gel in MES buffer.

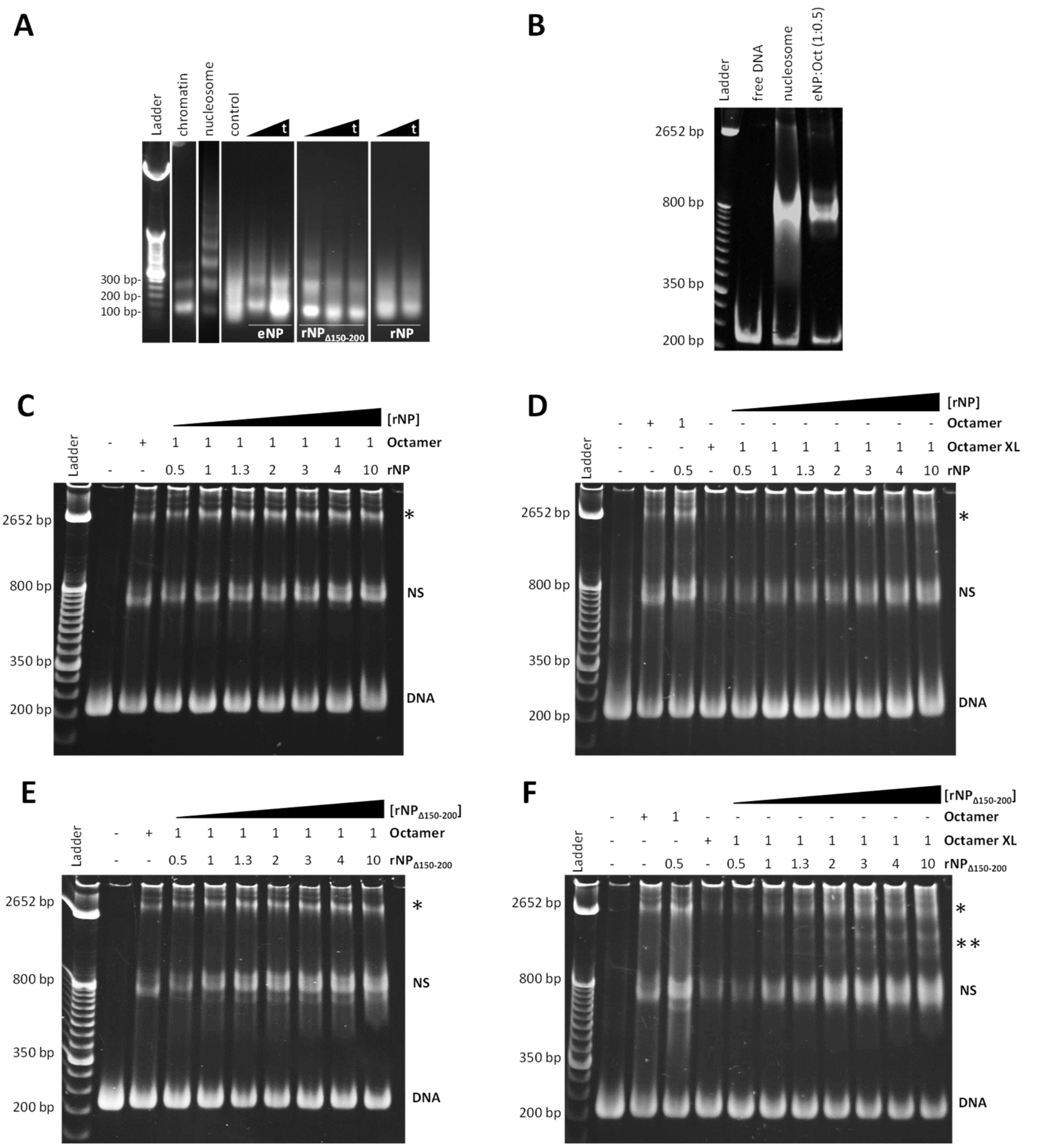

Figure S7. Nucleoplasmin (NP)-mediated nucleosome assembly depends on chaperone post-translational modification and on its C-terminal domain. (A) Micrococcal nuclease (MN) digestion of NP/Histone octamer (1/0.5 molar ratio) mixtures added to pBlueScript II plasmid (histone octamer/plasmid 1/0.8 µg/µg). Different digestion times (5,10 and 5,10,15 min) were taken for eNP, rNP$_{\Delta150\text{-}200}$ or rNP. MN digestion controls: H1 depleted erythrocyte chromatin (chromatin), salt reconstituted nucleosomes (nucleosome), plasmid /histone octamer (1/0.8 µg/µg) mixture (control). **(B)** Comparison of nucleosomes obtained by the salt dialysis method (nucleosome lane) and eNP-mediated assembly (eNP:Oct lane). Increasing rNP (**C** and **D**) or rNP$_{\Delta150\text{-}200}$ (**E** and **F**) concentrations were mixed with 0.4 µM native (**C** and **E**) or crosslinked **(D** and **F**) histone octamers, and incubated 1 h before the addition of 0.4 µM DNA. After 2h at room temperature and 30 min at 42 °C, samples were analyzed by 6% native PAGE and stained with SRBR Green. The NP/Octamer molar ratio used is noted above each lane. Bands marked with NS correspond to nucleosomes, and those with a single or double asterisk may be due to histone/DNA aggregates or to histone/DNA complexes generated upon transfer of crosslinked histone complexes.

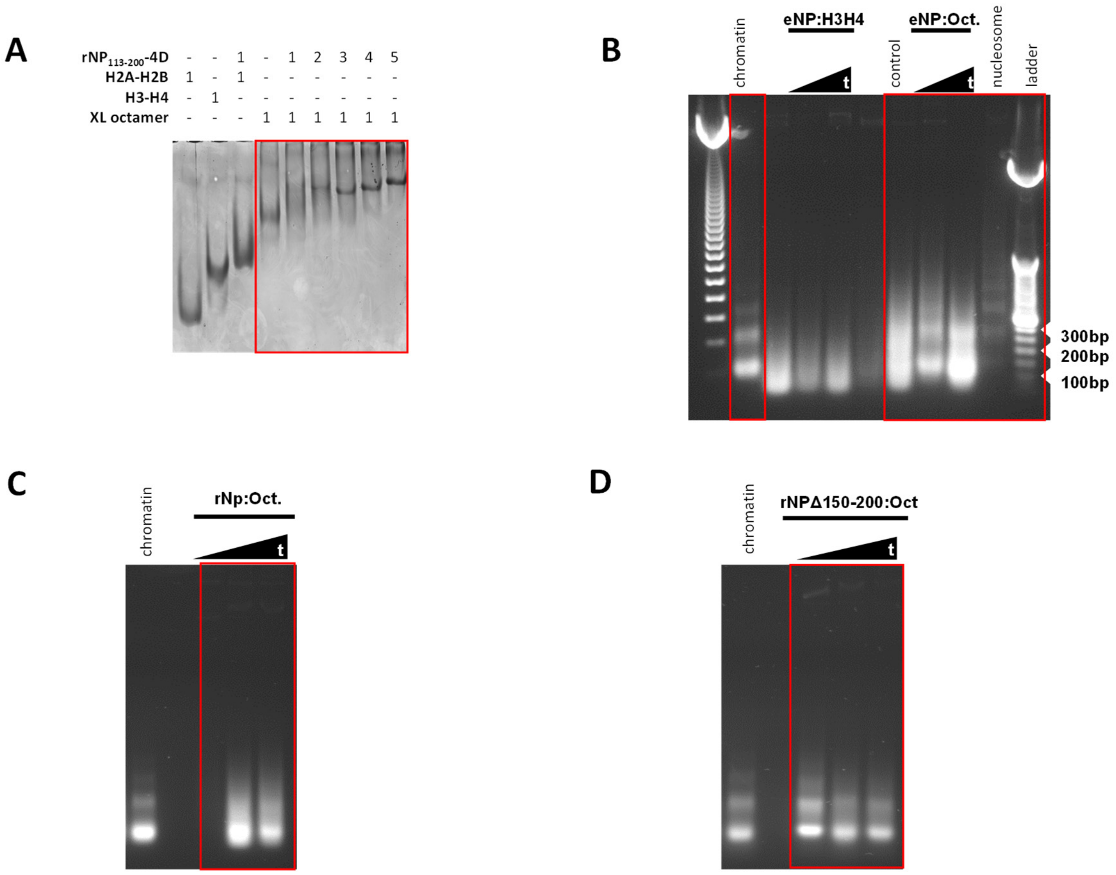

Figure S8. **Complete electrophoretic gel images.** Panel A is related to figure 5C. Only the lanes within the red box were used for the main figure and the others, present in the other gel of this panel, were cut off to save space. Panels B, C and D are related to Figure S7A. Only the lanes within the red boxes were used to compose panel S7A.

SUPPLEMENTARY REFERENCES

1 Fernandez-Rivero, N. *et al.* A Quantitative Characterization of Nucleoplasmin/Histone Complexes Reveals Chaperone Versatility. *Sci Rep* **6**, 32114, doi:10.1038/srep32114 (2016).

2 Hierro, A., Arizmendi, J. M., Banuelos, S., Prado, A. & Muga, A. Electrostatic interactions at the C-terminal domain of nucleoplasmin modulate its chromatin decondensation activity. *Biochemistry* **41**, 6408-6413 (2002).

3 Ramos, I. *et al.* The intrinsically disordered distal face of nucleoplasmin recognizes distinct oligomerization states of histones. *Nucleic Acids Res* **42**, 1311-1325, doi:10.1093/nar/gkt899 (2014).
